# Comparative effectiveness of levetiracetam, valproate and carbamazepine among elderly patients with newly diagnosed epilepsy: subgroup analysis of the randomized, unblinded KOMET study

**DOI:** 10.1186/s12883-016-0663-7

**Published:** 2016-08-23

**Authors:** Bernd Pohlmann-Eden, Anthony G. Marson, Matthias Noack-Rink, Francisco Ramirez, Azita Tofighy, Konrad J. Werhahn, Imane Wild, Eugen Trinka

**Affiliations:** 1 Epilepsy Program, Division of Neurology, Queen Elizabeth II Health Science Center, Dalhousie University, Halifax, Canada; 2 Brain Repair Center, Dalhousie University, Halifax, Canada; 3 Department of Molecular and Clinical Pharmacology, University of Liverpool, Liverpool, UK; 4 UCB Pharma, Monheim am Rhein, Germany; 5 UCB Pharma, Brussels, Belgium; 6 Department of Neurology, Paracelsus Medical University, Christian Doppler Klinik, and Centre for Cognitive Neuroscience, Salzburg, Austria

**Keywords:** Epilepsy, Elderly, Antiepileptic drug, Monotherapy, Levetiracetam

## Abstract

**Background:**

Few clinical trials have evaluated the efficacy and tolerability of antiepileptic drugs (AEDs) as initial monotherapy for elderly patients.

**Methods:**

This post-hoc subgroup analysis of data from an unblinded, randomized, 52-week superiority study (KOMET) compared the effectiveness of levetiracetam (LEV) with extended-release sodium valproate (VPA-ER) and controlled-release carbamazepine (CBZ-CR) as monotherapy in patients aged ≥ 60 years with newly diagnosed epilepsy. The physician chose VPA or CBZ as preferred standard treatment; patients were randomized to standard AEDs or LEV. The primary endpoint was time to treatment withdrawal. Results are exploratory, since KOMET was not powered for a subgroup analysis by age.

**Results:**

Patients (*n* = 308) were randomized to LEV (*n* = 48) or VPA-ER (*n* = 53) in the VPE-ER stratum or to LEV (*n* = 104) or CBZ-CR (*n* = 103) in the CBZ-CR stratum. Mean age was 69.6 years, range 60.2–89.9 years (intention-to-treat population *n* = 307). Time to treatment withdrawal hazard ratio [HR] (95 % confidence interval [CI]) for LEV vs. standard AEDs was 0.44 (0.28–0.67); LEV vs. VPA-ER: 0.46 (0.16–1.33); LEV vs. CBZ-CR: 0.45 (0.28–0.72). Twelve-month withdrawal rates were: LEV vs. standard AEDs, 20.4 vs. 38.7 %; LEV vs. VPA-ER, 10.4 vs. 23.1 %; LEV vs. CBZ-CR, 25.0 vs. 46.6 %. Time to first seizure was similar between LEV and standard AEDs (HR: 0.92, 95 % CI: 0.63–1.35), LEV and VPA-ER (0.77, 0.38–1.56), and LEV and CBZ-CR (1.02, 0.64–1.63). Adverse events were reported by 76.2, 67.3, and 82.5 % of patients for LEV, VPA-ER, and CBZ-CR, respectively. Discontinuation rates due to AEs were 11.3, 10.2, and 35.0 % for LEV, VPA-ER, and CBZ-CR, respectively.

**Conclusions:**

Time to treatment withdrawal was longer with LEV compared with standard AEDs. This finding was driven primarly by the result in the CBZ-CR stratum, which in turn was likely due to the more favorable tolerability profile of LEV. Results of this post-hoc analysis suggest that LEV may be a suitable option for initial monotherapy for patients aged ≥ 60 years with newly diagnosed epilepsy.

**Trial registration:**

ClinicalTrials.gov: NCT00175903; September 9, 2005.

**Electronic supplementary material:**

The online version of this article (doi:10.1186/s12883-016-0663-7) contains supplementary material, which is available to authorized users.

## Background

The increased incidence of new-onset epilepsy in the elderly has been recognized for some time [1–3]. Given the rapidly aging population, epilepsy in the elderly is likely to become one of the most frequent forms of epilepsy encountered in clinical practice.

Characteristics of epilepsy, such as etiology, clinical manifestations, and electroencephalogram (EEG) findings differ between elderly and younger populations [4–7]. Among the elderly, cerebrovascular disease is the leading identifiable cause of epilepsy; others include trauma, dementia and brain tumours, typically gliomas, meningiomas, and brain metastases [6, 8–10]. However, in a large number of cases no obvious etiology can be identified [11]. New-onset seizures in the elderly are typically focal, with or without secondary generalization, reflecting their regional etiology and most often their underlying structural cause [12, 13]. Diagnosis of focal seizures with or without impairment of consciousness can be challenging in the elderly, since aura phenomena or automatisms are less frequent than in younger individuals. Also the period of postictal confusion can be much prolonged [10, 12].

The choice of antiepileptic drug (AED) for elderly patients is particularly challenging [4, 5, 12, 14], notably due to age-related physiological changes which affect drug pharmacokinetics and pharmacodynamics [15]. The elderly typically have reduced capacity to metabolize drugs, to excrete drugs via the kidneys, and reduced plasma protein drug binding due to reduced concentrations of albumin [16, 17]. In addition, there is a correlation between increasing age and the incidence of adverse drug reactions [18]. AEDs that induce or inhibit the expression of CYP450 enzymes may affect the metabolism of many commonly prescribed drugs resulting in clinically relevant drug-drug interactions [4, 5, 8, 19, 20]—this is especially important among elderly patients who frequently require polytherapy for comorbidities. In a retrospective study in veterans (≥66 years) with epilepsy, almost half of this population were receiving an AED that potentially interacted with their existing medication, most commonly cardiovascular drugs [21].

Despite the important medical need for effective and well-tolerated AEDs for elderly patients, very few randomized controlled clinical trials have been conducted. This is predominantly due to challenges inherent in conducting monotherapy trials in epilepsy [22]; difficulties in recruiting elderly patients, the high number of comorbidities and diagnostic complexity also contribute to the paucity of clinical evidence [9, 23, 24]. Based on these observations, post-hoc analyses of data from large-scale clinical studies may be warranted. The Keppra vs. Older Monotherapy in Epilepsy Trial (KOMET) was a large-scale, Phase IV trial conducted to compare the effectiveness of levetiracetam (LEV) with either extended-release sodium valproate (VPA-ER) or controlled-release carbamazepine (CBZ-CR), according to physician choice, in patients aged ≥ 16 years with newly diagnosed epilepsy [25]. Like its predecessor, the Standard and New Antiepileptic Drugs (SANAD) trial [26, 27], KOMET was a pragmatic, randomized trial and, as such, it was not blinded. Treatment choice—either VPA-ER or CBZ-CR and subsequent randomization—was based on investigators’ judgment, which in clinical practice is mainly based on clarity of diagnosis, seizure type, and patient characteristics. This report presents the results of a post-hoc subgroup analysis conducted to compare the effectiveness of LEV with VPA-ER and CBZ-CR among patients aged ≥ 60 years who participated in KOMET.

## Methods

KOMET (N01175; NCT00175903) was a multicenter, unblinded, randomized, 52-week, controlled superiority trial with a two-parallel-group design. It was carried out in a community setting at 269 centers across 23 European countries and Australia between February 2005 and October 2007.

Trial design, methodology, and statistical analysis have been published [25]. Briefly, patients aged ≥ 16 years who had experienced two or more unprovoked seizures in the previous 2 years with at least one during the previous 6 months were included. At screening, the investigator decided whether VPA or CBZ would be the standard first-line treatment. Within the VPA stratum, patients were randomized (1:1) to treatment with LEV (UCB Pharma, Belgium) or VPA-ER (Sanofi-Aventis, France). Within the CBZ stratum, patients were randomized (1:1) to treatment with LEV or CBZ-CR (Novartis, Switzerland). Starting doses (LEV 500 mg/day, VPA-ER 500 mg/day, CBZ-CR 200 mg/day) were up-titrated over 2 weeks to the target doses (LEV 1000 mg/day, VPA-ER 1000 mg/day, CBZ-CR 600 mg/day). Doses could be increased to a maximum of LEV 3000 mg/day, VPA-ER 2000 mg/day, and CBZ-CR 1600 mg/day, according to the clinician’s judgement.

All participants provided written informed consent before entering the study. The study was approved by local ethics committees for every study center. An additional file shows the full names of all Institutional Review Boards (see Additional file 1). The study was conducted in accordance with International Conference on Harmonisation Good Clinical Practice guidelines and the Declaration of Helsinki.

### Patients

Based on the United Nations’ definition of the elderly [28], this post-hoc subgroup analysis included all patients who were aged ≥ 60 years at trial entry. The 60-year cut-off point for age was also chosen to allow for sufficiently large patient numbers in each of the treatment groups.

### Outcome measures

The primary outcome measure was time to withdrawal from study medication (treatment withdrawal) calculated from randomization to the day after the last intake of study medication. Secondary outcome measures were time to first seizure calculated from randomization; and treatment withdrawal and seizure freedom rates at 6 and 12 months. LEV was compared with standard AEDs (combined VPA and CBZ strata), and with VPA-ER and CBZ-CR within the individual strata. Results for the VPA and CBZ strata excluded patients with unclassified seizure types. Results for each stratum are also reported for subgroups with only focal or only generalized seizures (excluding unclassified, unknown or mixed seizure types). Tolerability was evaluated by documenting treatment-emergent adverse events (TEAEs), their intensity classified as judged by the investigator (mild, moderate, or severe) and seriousness.

### Statistical analysis

All results are exploratory since KOMET was not powered for a subgroup analysis by age. The intention-to-treat (ITT) population included all randomized patients, regardless of actual drug intake. The safety population consisted of all patients who received one or more doses of study medication, including those with unknown drug intake. Patients who were randomized but not treated, and those who did not give informed consent, were excluded from the safety population.

Kaplan-Meier survival curves were plotted for time to treatment withdrawal and time to first seizure. For the time to treatment withdrawal analysis, all treated patients who withdrew from the study prior to Day 365 were considered as having the event. Patients who completed the study or withdrew after Day 365 were censored at Day 365 or at completion of the study (for those who completed just prior to Day 365). Untreated (but randomized) patients were censored at Day 1 (one day after randomization). For the time to first seizure analysis, patients with no reported seizure during the 12-month treatment period were censored at the date of last intake of study medication, date of early termination, date of Week 52 visit, or Day 364, whichever was earliest. For the time to treatment withdrawal and time to first seizure analyses, no further adjustments for dropouts or missing data were required, as these patients were censored accordingly.

Time to treatment withdrawal and time to first seizure were analysed using a Cox’s proportional hazards regression model including treatment and classification of epilepsy. The treatment effect hazard ratio (HR) was described using two-sided 95 % confidence intervals (CIs); a HR of < 1 favored LEV, while a HR of > 1 favoured standard AEDs. Kaplan-Meier survival curves were plotted for time to treatment withdrawal due to an AE and calculated from randomization. Since the primary focus of the post-hoc analysis was on age, a model was derived from the entire KOMET population, of time to treatment withdrawal that included the interaction of treatment and age, and the statistical significance of this interaction was used as supporting evidence.

## Results

**Fig. 1:**
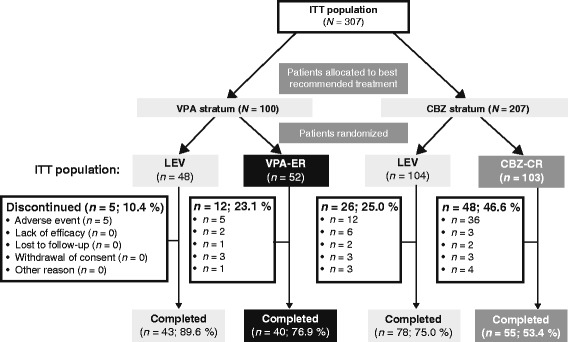
Patient disposition

Overall, 1698 patients were randomized to KOMET, of whom 308 were aged ≥ 60 years and therefore included in this analysis (Fig. 1). VPA was deemed by the treating physicians to be standard treatment for 101 patients who were subsequently randomized to treatment with either LEV (*n* = 48) or VPA-ER (*n* = 53). Similarly patients allocated to the CBZ stratum (*n* = 207) by physicians were subsequently randomised to treatment with either LEV (*n* = 104) or CBZ-CR (*n* = 103). One patient randomized to VPA-ER was excluded from the ITT population due to no documented informed consent.

At 12 months, retention rates in the VPA stratum were 89.6 % in the LEV group and 76.9 % in the VPA-ER group. Corresponding values in the CBZ-CR stratum were 75.0 % and 53.4 % in the LEV and CBZ-CR treatment arms, respectively (Fig. 1).

Baseline demographic characteristics were similar for the LEV and standard AED groups (Table 1). Within the VPA stratum 36/100 patients (36.0 %) had generalized seizures only, and within the CBZ stratum 187/207 patients (90.3 %) had focal seizures only. The majority of patients had epilepsy either due to an unknown cause (LEV 56.6 %, standard AEDs 57.4 %) or cerebrovascular accident (LEV 27.0 %, standard AEDs 30.3 %). The majority were also receiving drugs for the management of cardiovascular disorders (LEV 69.7 %, standard AEDs 75.5 %) including angiotensin converting enzyme inhibitors, statins and beta-blockers.

**Table 1:** Baseline demographics and epilepsy characteristics (intent-to-treat population)

	Overall		VPA stratum		CBZ stratum	
	LEV (*n* = 152)	Standard AEDs (*n* = 155)	LEV (*n* = 48)	VPA-ER (*n* = 52)	LEV (*n* = 104)	CBZ-CR (*n* = 103)
Age, years, mean (SD)	69.5 (6.2)	69.7 (6.4)	71.1 (6.8)	70.4 (6.5)	68.8 (5.9)	69.3 (6.4)
Gender, *n* (%)						
Men	85 (55.9)	87 (56.1)	25 (52.1)	33 (63.5)	60 (57.7)	54 (52.4)
Women	67 (44.1)	68 (43.9)	23 (47.9)	19 (36.5)	44 (42.3)	49 (47.6)
Number of seizures in the last 2 years^a^, median (Q1–Q3) [*n*]	4 (2–6) [150]	4 (2–8) [149]	3 (2–6) [47]	3 (2–5) [51]	4 (2–8) [102]	4 (3–10) [99]
Epilepsy duration^b^, years, median (Q1–Q3)	0.70 (0.27–2.95)	0.70 (0.30–2.12)	0.79 (0.20–2.24)	0.73 (0.26–1.84)	0.63 (0.28–3.15)	0.70 (0.32–2.55)
Seizure type^c^, *n* (%)						
Simple focal	30 (19.7)	32 (20.6)	5 (10.4)	4 (7.7)	25 (24.0)	28 (27.2)
Complex focal	62 (40.8)	62 (40.0)	15 (31.3)	11 (21.2)	47 (45.2)	51 (49.5)
SG or tonic clonic	84 (55.3)	89 (57.4)	30 (62.5)	38 (73.1)	54 (51.9)	51 (49.5)
Other^d^	4 (2.6)	4 (2.6)	3 (6.3)	3 (5.8)	1 (1.0)	1 (1.0)
Unclassified^e^	5 (3.3)	3 (1.9)	4 (8.3)	3 (5.8)	1 (1.0)	0

*Abbreviations*: *AED* antiepileptic drug, *CBZ* carbamazepine, *CBZ-CR* controlled-release carbamazepine, *LEV* levetiracetam, *SG* secondarily generalised, *VPA* sodium valproate, *VPA*-*ER* extended-release sodium valproate

^a^Overall: LEV, *n* = 149; standard AEDs, *n* = 150. CBZ stratum: LEV, *n* = 102; CBZ-CR, *n* = 99. VPA stratum: LEV, *n* = 47; VPA-ER, *n* = 51

^b^Time since first seizure

^c^Patients are counted once in each type of seizure experienced

^d^Including absence, myoclonic, clonic, tonic and atonic seizures

^e^Including unclassified, unable to clarify and unknown seizure types

### LEV versus standard AEDs

**Fig. 2:**
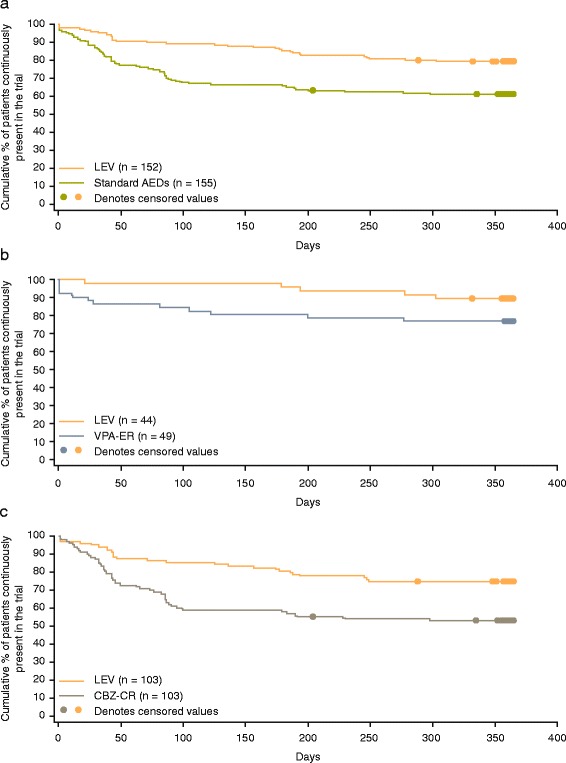
Kaplan-Meier survival curves for the time to treatment withdrawal (intent-to-treat population). Legend: **a** LEV vs. standard AEDs; **b** LEV vs. VPA-ER in the VPA stratum; and **c** LEV vs. CBZ-CR in the CBZ stratum. Patients with unclassified seizure types were excluded from analyses for the VPA and CBZ strata. Abbreviations: *AED* antiepileptic drug; *CBZ* carbamazepine; *CBZ-CR* controlled-release carbamazepine; *LEV* levetiracetam; *VPA* sodium valproate; *VPA-ER* extended-release sodium valproate

**Fig. 3:**
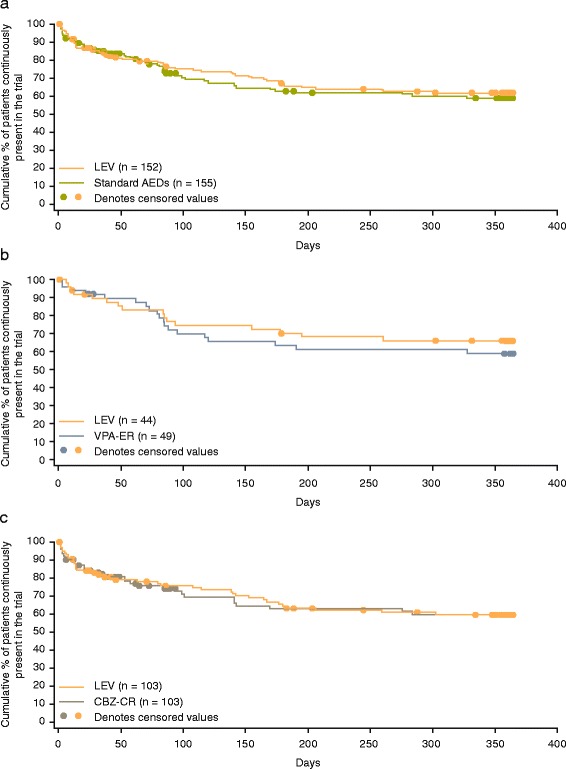
Kaplan-Meier survival curves for the time to first seizure (intent-to-treat population). Legend: **a** LEV vs. standard AEDs; **b** LEV vs. VPA-ER in the VPA stratum; and **c** LEV vs. CBZ-CR in the CBZ stratum. Patients with unclassified seizure types were excluded from analyses for the VPA and CBZ strata. Abbreviations: *AED* antiepileptic drug; *CBZ* carbamazepine; *CBZ-CR* controlled-release carbamazepine; *LEV* levetiracetam; *VPA* sodium valproate; *VPA-ER* extended-release sodium valproate

Time to treatment withdrawal (primary endpoint) was longer in patients treated with LEV compared with those treated with standard AEDs (HR 0.44, 95 % CI 0.28–0.67) (Fig. 2a); similarly, treatment withdrawal rates at 6 and 12 months were also lower for LEV-treated patients (Table 2). Time to first seizure (HR 0.92, 95 % CI 0.63–1.35) [Fig. 3a] and seizure freedom rates were comparable in both groups (Table 2). Note: HR of < 1 favors LEV. In the analysis of the whole KOMET dataset, no difference was observed in time to treatment withdrawal between LEV and standard AEDs [25]. However a significant interaction between treatment and age (*p* = 0.002) was identified, indicating that treatment effect differs with age, supporting the results of the primary endpoint in the elderly population.

**Table 2:** Treatment withdrawal and seizure freedom rates for levetiracetam and standard antiepileptic drugs (intent-to-treat population)

	Levetiracetam (*n* = 152)	Standard antiepileptic drugs (*n* = 155)
Treatment withdrawal rate, % (95 % CI)		
6 months	14.5 (9.8–21.1)	34.2 (27.3–42.2)
12 months	20.4 (14.8–27.7)	38.7 (31.6–46.9)
Seizure freedom rate, % (95 % CI)		
6 months	65.6 (57.1–72.8)	62.9 (53.7–70.7)
12 months	61.8 (53.2–69.3)	59.1 (49.8–67.3)

*Abbreviations*: *CI* confidence interval

### LEV versus VPA-ER

A trend towards longer time to treatment withdrawal was observed in patients in the LEV group compared with those in the VPA-ER group (HR 0.46, 95 % CI 0.16–1.33) (Fig. 2b). Correspondingly, estimated treatment withdrawal rates were higher for patients treated with VPA-ER than LEV at both 6 and 12 months (Table 3), though the 95 % CIs did not support a difference between these groups. Time to first seizure was similar in the LEV and VPA-ER groups (HR 0.77, 95 % CI 0.38–1.56) (Fig. 3b), as were the estimated 6- and 12-month seizure freedom rates (Table 3). Treatment withdrawal and seizure freedom rates reported in patients who only experienced generalized seizures were similar to those seen for all patients in the VPA stratum (Table 3). In the analysis of the whole KOMET dataset, no evidence of an interaction between treatment and age was found between the VPA-ER and LEV groups (*p* = 0.171), in agreement with the analysis in the elderly population.

**Table 3:** Treatment withdrawal and seizure freedom rates (Kaplan-Meier estimates) for sodium valproate and carbamazepine strata (intent-to-treat population)^a^

Sodium valproate stratum		LEV	VPA-ER
		All seizure types *n* = 48	All seizure types *n* = 52
		Generalized seizures only *n* = 14	Generalized seizures only *n* = 22
Treatment withdrawal rate, % (95 % CI)			
6 months	All seizure types	4.2 (1.1–15.7)	19.2 (10.8–32.8)
	Generalized seizures only	7.1 (1.0–40.9)	18.2 (7.2–41.5)
12 months	All seizure types	10.4 (4.5–23.2)	23.1 (13.8–37.0)
	Generalized seizures only	14.3 (3.8–46.1)	22.7 (10.2–46.3)
Seizure freedom rate, % (95 % CI)			
6 months	All seizure types	70.4 (55.1–81.3)	63.4 (47.8–75.4)
	Generalized seizures only	69.6 (37.8–87.4)	63.3 (38.1–80.6)
12 months	All seizure types	66.0 (50.5–77.6)	59.0 (43.5–71.6)
	Generalized seizures only	–	52.8 (28.9–72.0)
Carbamazepine stratum		LEV	CBZ-CR
		All seizure types *n* = 104	All seizure types *n* = 103
		Focal seizures only *n* = 93	Focal seizures only *n* = 94
Treatment withdrawal rate, % (95 % CI)			
6 months	All seizure types	19.2 (12.9–28.2)	41.8 (32.9–51.9)
	Focal seizures only	18.3 (11.8–27.8)	40.4 (31.3–51.1)
12 months	All seizure types	25.0 (17.8–34.5)	46.6 (37.6–56.7)
	Focal seizures only	24.7 (17.2–34.8)	45.8 (36.4–56.4)
Seizure freedom rate, % (95 % CI)			
6 months	All seizure types	63.3 (52.6–72.1)	63.2 (51.4–72.8)
	Focal seizures only	59.3 (48.0–68.9)	63.1 (51.0–73.1)
12 months	All seizure types	59.8 (49.1–69.0)	59.8 (47.7–69.9)
	Focal seizures only	55.5 (44.2–65.4)	61.3 (49.0–71.5)

*Abbreviations*: *CBZ-CR* controlled-release carbamazepine, *CI* confidence interval, *LEV* levetiracetam, *VPA-ER* extended-release sodium valproate

^a^For the sodium valproate stratum, data are presented for all patients (intent-to-treat population excluding unclassified seizure types) and those with generalized seizures only (intent-to-treat population excluding unclassified, unknown and mixed seizure types). For the carbamazepine stratum, data are presented for all patients (intent-to-treat population excluding unclassified seizure types) and those with focal seizures only (intent-to-treat population excluding unclassified, unknown and mixed seizure types)

### LEV versus CBZ-CR

Time to treatment withdrawal was longer in patients treated with LEV compared with those treated with CBZ-CR (HR 0.45, 95 % CI 0.28–0.72) (Fig. 2c). Estimated 6- and 12-month treatment withdrawal rates were higher for patients treated with CBZ-CR than LEV (Table 3). Time to first seizure was similar in the LEV and CBZ-CR groups (HR 1.02, 95 % CI 0.64–1.63) (Fig. 3c), as were the estimated 6- and 12-month seizure freedom rates (Table 3). Treatment withdrawal and seizure freedom rates reported in patients who only experienced focal seizures were similar to those seen for all patients in the CBZ stratum (Table 3). In the analysis of time to withdrawal in the whole KOMET dataset, the interaction between treatment and age was significant (*p* = 0.009), supporting the comparison of LEV and CBZ-CR in the elderly population.

### Safety and tolerability

At least one TEAE was reported by 76.2 % of patients treated with LEV (both strata combined) compared with VPA-ER (67.3 %) and CBZ-CR (82.5 %) [Table 4]. Higher incidences of severe AEs were reported in the LEV (20.5 %) and CBZ-CR (17. 5 %) groups than in the VPA-ER group (8.2 %). Although serious AEs were more frequently reported by patients treated with LEV (18.5 %) than CBZ-CR (10.7 %) or VPA-ER (4.1 %), drug-related serious AEs had a comparably low incidence in all groups (2.0 %, 2.9 %, and 2.0 %, respectively).

**Table 4:** Summary of treatment-emergent adverse events (safety population)

	All patients			
	LEV (*n* = 151)	Standard AEDs (*n* = 152)	VPA-ER (*n* = 49)	CBZ-CR (*n* = 103)
Summary of adverse events, *n* (%)				
≥1 TEAE	115 (76.2)	118 (77.6)	33 (67.3)	85 (82.5)
Drug-related AEs	70 (46.4)	93 (61.2)	29 (59.2)	64 (62.1)
Severe AEs^a^	31 (20.5)	22 (14.5)	4 (8.2)	18 (17.5)
Serious AEs	28 (18.5)	13 (8.6)	2 (4.1)	11 (10.7)
Serious drug-related AEs	3 (2.0)	4 (2.6)	1 (2.0)	3 (2.9)
AEs leading to discontinuation	17 (11.3)	41 (27.0)	5 (10.2)	36 (35.0)
Deaths	2 (1.3)	1 (0.7)	0	1 (1.0)
Drug-related TEAEs reported by ≥ 5 % of patients in any treatment group, *n* (%)				
Fatigue	17 (11.3)	33 (21.7)	9 (18.4)	24 (23.3)
Dizziness	10 (6.6)	16 (10.5)	0	16 (15.5)
Somnolence	8 (5.3)	9 (5.9)	2 (4.1)	7 (6.8)
Headache	8 (5.3)	7 (4.6)	0	7 (6.8)
Nausea	4 (2.6)	11 (7.2)	1 (2.0)	10 (9.7)
Weight gain	4 (2.6)	11 (7.2)	6 (12.2)	5 (4.9)
Tremor	1 (0.7)	9 (5.9)	7 (14.3)	2 (1.9)
Constipation	1 (0.7)	6 (3.9)	0	6 (5.8)
Rash	0	8 (5.3)	0	8 (7.8)

*Abbreviations*: *AE* adverse event, *AED* antiepileptic drug, *CBZ* carbamazepine, *CBZ-CR* controlled-release carbamazepine, *LEV* levetiracetam, *TEAE* treatment-emergent adverse event, *VPA* sodium valproate, *VPA-ER* extended-release sodium valproate

^a^Severe AEs were those affecting the patient’s ability to work normally or to carry out usual activities, and those of definite clinical consequence

TEAEs most commonly reported (≥10 % of patients) were fatigue (11.3 %) with LEV; fatigue (18.4 %), tremor (14.3 %), and weight gain (12.2 %) with VPA-ER; and fatigue (23.3 %) and dizziness (15.5 %) with CBZ-CR (Table 4).

**Fig. 4:**
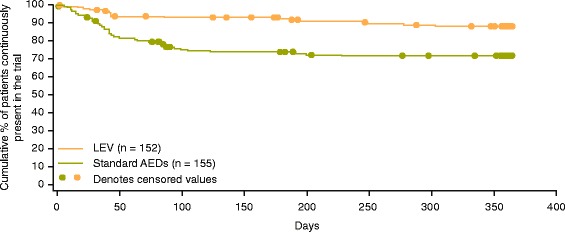
Kaplan-Meier survival curves for the time to withdrawal due to an adverse event (intent-to-treat population). Abbreviations: *AED* antiepileptic drug; *LEV* levetiracetam

Time to treatment withdrawal due to AEs was longer for LEV than for standard AEDs (HR 0.36, 95 % CI 0.20–0.63) (Fig. 4). In the safety population, more patients treated with standard AEDs discontinued treatment due to AEs than those treated with LEV (27.0 % vs. 11.3 %); mostly due to the higher proportion of withdrawals in the CBZ-CR group (35.0 %) (Table 4).

Three deaths were reported; two patients treated with LEV (head injury sustained in road traffic accident; radiation injury) and one treated with CBZ-CR (acute myocardial infarction). None of the deaths were considered to be related to study medication.

## Discussion

This post-hoc subgroup analysis of data from KOMET, an unblinded, randomized trial [25], compared the effectiveness of LEV with that of standard AEDs among patients aged ≥ 60 years with newly diagnosed epilepsy. Consistent with existing data [13, 29, 30] and in keeping with acquired focal brain pathology in the elderly, the majority of patients included in this analysis experienced focal seizures, of which complex focal (automotor) seizures were more frequent than simple focal seizures. The most frequent identifiable causes of epilepsy were cerebrovascular, also consistent with previous observations [6, 8, 10, 14, 31]. The majority of patients were allocated to CBZ as standard treatment, although as many as one-third were allocated to the VPA stratum. It is important to note that KOMET was a pragmatic trial, and that choice of treatment was not determined by protocol, but by the treating physicians. Consequently, not all patients may have received what is considered standard treatment; indeed, robust evidence for a standard treatment is lacking in the elderly. Furthermore, diagnosis of epilepsy and classification of seizure type in the elderly population present significant challenges. Among patients thought to have generalized seizures, some may actually have had secondary generalized seizures and arguably, once again, were not allocated standard treatment. At the time KOMET was conducted, VPA was commonly prescribed for older people due to its broad therapeutic spectrum and straightforward dosing schedule [6, 32–34]. In contrast, CBZ can be difficult to use in the elderly because as an enzyme inducer, it has strong potential for drug-drug interactions, adverse impact on bone health [19, 33, 35], lipids and cardiovascular risk [36, 37], and influence on cardiac conduction systems [6]; all important concerns in this population.

Overall, LEV showed an advantage over standard AEDs in the elderly subpopulation, as demonstrated by a longer time to treatment withdrawal. The difference was driven predominantly by the finding in the CBZ stratum, as shown in the analysis of the individual strata. While time to treatment withdrawal was longer with LEV compared with CBZ-CR in the CBZ stratum, this was not the case in the VPA stratum. However, patients treated with LEV showed a potential advantage over those treated with VPA-ER in that the proportion of patients who withdrew from treatment at 12 months was greater with VPA-ER than with LEV (22.7 % vs. 14.3 % for patients with generalized seizures only; 23.1 % vs. 10.4 % for patients with all types of seizures). Treatment withdrawal rates at 12 months were also greater for elderly patients treated with CBZ-CR compared with those treated with LEV in the CBZ stratum and for standard AEDs in the overall comparison with LEV. Analysis of the interaction between treatment and age in time to treatment withdrawal using data from the entire KOMET population supported the results observed in this elderly subpopulation. A significant interaction between treatment and age was identified in the overall comparison, suggesting that the response to LEV or standard AEDs did indeed differ according to age. The interaction was also significant in the CBZ stratum, but not in the VPA stratum, once again reflecting the results of this subgroup analysis in elderly patients.

Time to first seizure analysis suggested similarity between LEV and standard AEDs, and between LEV and CBZ-CR or VPA-ER in the individual strata. Approximately one-third of the elderly patients who were allocated to the VPA stratum experienced generalized seizures only, predominantly tonic-clonc; results for this group of patients were similar to the overall results for the VPA stratum. Correspondingly, results for patients allocated to the CBZ stratum who only experienced focal seizures were similar to the overall results for this stratum.

With regard to tolerability, the time to treatment withdrawal due to AEs was longer in patients treated with LEV compared with standard AEDs. In particular, the discontinuation rate for patients randomized to CBZ-CR was higher than that for patients taking LEV or VPA-ER. However, it should be noted that the initial target dose of CBZ-CR (600 mg/day) may have been too high and the up-titration schedule may have been too rapid in this elderly population, resulting in the higher discontinuation rate [5]. Comparison of the discontinuation rates due to AEs in the CBZ-CR group in KOMET overall (18.8 %) and this subgroup analysis (35.0 %) indicates that the target dose of CBZ-CR was tolerated better by younger patients. Differences in withdrawal rates due to AEs between KOMET overall and the elderly subgroup were less pronounced for LEV (8.3 % vs. 11.3 %; safety population) and VPA-ER (4.7 % vs. 10.2 %; safety population). Nonetheless, these observations suggest that the difference between LEV and standard AEDs in the overall time to treatment withdrawal may be largely explained by differences in tolerability.

The main trial, KOMET, included 1688 patients (intention-to-treat population) with an overall mean age of 41 years [25]. Regarding time to treatment withdrawal, KOMET reported that LEV monotherapy was not superior to standard AEDs overall (HR 0.90, 95 % CI 0.74–1.08) or in the individual strata vs. VPA-ER (HR 1.02, 95 % CI 0.74–1.41) and CBZ-CR (HR 0.84, 95 % CI 0.66–1.07). In contrast, in this subgroup analysis of elderly patients, time to treatment withdrawal was longer for LEV compared with standard AEDs (HR 0.44, 95 % CI 0.28–0.67) and CBZ-CR (HR 0.45, 95 % CI 0.28–0.72), but not VPA-ER (HR 0.46, 95 % CI 0.16–1.33). Conversely, time to first seizure was longer for standard AEDs compared with LEV in KOMET overall (HR 1.20, 95 % CI 1.03–1.39), but not in the elderly subgroup (HR 0.92, 95 % CI 0.63–1.35).

Interpretation of these findings should take into account that this was a post-hoc subgroup analysis of data from a larger trial. In considering these results, it should be noted that approximately twice as many elderly patients were assigned to the CBZ stratum vs. the VPA stratum. As a pragmatic trial, KOMET had a number of limitations, including patient selection and treatment allocation at the discretion of the physician, and unblinded treatment. Another major limitation is the lack of neuroimaging information with regard to underlying etiology, which likely has a major prognostic impact in this specific epilepsy population [38]. We challenge the term Epilepsy in the Elderly (EE), which refers to people older than 60 years. The term is likely to be conceptually irrelevant for disease management and prognosis [39], since biological age, as reflected in imaging findings, is more important than chronological age. In a recent prospective new-onset population, the underlying MRI-proven lesions were not significantly different in the EE group compared with patients aged between 50 and 60 years [39].

To date, five randomized, controlled trials of AED monotherapy have been conducted in elderly patients with newly diagnosed epilepsy [30, 31, 40–42]. In general, results have indicated that the AEDs tested in the trials have comparable efficacy in this population, but differing tolerability profiles. Four trials reported comparable efficacy between LTG and CBZ in terms of time to first seizure [40] or seizure freedom [30, 41, 42]. However, retention rates were higher for LTG than CBZ in the four trials, largely attributed to better tolerability with LTG. The Study on the Treatment of Elderly Patients with Older and Newer antiEpileptic drugs (STEP-ONE) trial was the first prospective, randomized, double-blind trial to compare LEV and LTG with CBZ-CR in elderly patients with newly diagnosed epilepsy. The results indicated that the efficacy of LEV monotherapy was similar to that of CBZ-CR, while tolerability was superior, leading to significantly greater effectiveness in terms of retention rate [31]. Our findings from this subgroup analysis of the KOMET study are in agreement with those from STEP-ONE, and provide further supporting evidence for the use of LEV monotherapy for elderly patients with newly diagnosed epilepsy.

## Conclusions

In this post-hoc analysis of data from KOMET, time to treatment withdrawal was longer with LEV than with standard AEDs. Since time to treatment withdrawal was similar between LEV and VPA-ER, the overall result was driven mainly by the results in the CBZ-CR stratum. Patients remained in the LEV treatment arm longer than those in the CBZ-CR arm, most likely because of the better tolerability of LEV. While the tolerability of CBZ-CR could have been improved by using a lower starting dose and slower up-titration, the long-term use of CBZ-CR in the elderly population is problematic in light of its enzyme-inducing properties. The results of this analysis are in agreement with those of the randomized, double-blind STEP-ONE trial and several prospective, observational studies. Consequently, LEV may be considered a suitable option as initial monotherapy for individuals aged 60 years or above with newly diagnosed epilepsy.

## Additional file

Additional file 1: Full names of all Institutional Review Boards that approved the KOMET protocol. (XLSX 28 kb)

## Abbreviations

AE: Adverse event
AED: Antiepileptic drug
CBZ: Carbamazepine
CBZ-CR: Controlled-release carbamazepine
CI: Confidence interval
EE: Epilepsy in the Elderly
EEG: Electroencephalogram
HR: Hazard ratio
ITT: Intention-to-treat
KOMET: Keppra vs. Older Monotherapy in Epilepsy Trial
LEV: Levetiracetam
LTG: Lamotrigine
SANAD: Standard and New Antiepileptic Drugs
STEP-ONE: Study on the Treatment of Elderly Patients with Older and Newer antiEpileptic Drugs
TEAE: Treatment-emergent adverse event
VPA: Sodium valproate
VPA-ER: Extended-release sodium valproate
